# Is ‘mainstreaming AYUSH’ the right policy for Meghalaya, northeast India?

**DOI:** 10.1186/s12906-015-0818-x

**Published:** 2015-08-18

**Authors:** Sandra Albert, John Porter

**Affiliations:** Indian Institute of Public Health, Lawmali, Pasteur Hill, 793 001 Shillong, Meghalaya India; Public Health Foundation of India, New Delhi, India; London School of Hygiene & Tropical Medicine, London, UK

**Keywords:** Health policy, Health systems, Indigenous medicine, Medical pluralism, Traditional medicine

## Abstract

**Background:**

National policy on medical pluralism in India encourages the mainstreaming of AYUSH (Ayurveda, Yoga, Unani, Siddha, and Homeopathy) systems and the revitalization of local health traditions (LHT). In Meghalaya state in the northeast, the main LHT is its indigenous tribal traditional medicine. This paper presents the perceptions of tribal medicine and of AYUSH systems among various policy actors and locates the tribal medicine of Meghalaya within the policy on medical pluralism currently being implemented in the state, a region that is ethnically and culturally different and predominantly inhabited by indigenous peoples.

**Methods:**

A stakeholder mapping exercise identified appropriate policy actors and 46 in-depth interviews were conducted with policy makers, doctors, academics, members of healer associations and elders of the community. A further 44 interviews were conducted with 24 Khasi and 20 Garo traditional healers. Interview data were supplemented with document analysis and observations. Qualitative data were analyzed using thematic content analysis that incorporated elements of grounded theory.

**Results:**

In Meghalaya there is high awareness and utilization of tribal medicine, but no visible efforts by the public sector to support or engage with healers. The AYUSH systems in contrast had little local acceptance but promotion of these systems has led to a substantial increase in AYUSH doctors, particularly homeopaths, in rural areas. Policy actors outside the health department saw an important role for tribal medicine due to its popularity, local belief in its efficacy and its cultural resonance. The need to engage with healers to enhance referral, training, documentation and research of tribal medicine was made.

**Conclusions:**

The wide acceptance of tribal medicine suggests that tribal medicine needs to be supported. The results of the study question the process of the implementation of the ‘mainstreaming AYUSH’ policy for Meghalaya and highlight the importance of contextualizing health policy within the local culture. A potential role for Health Policy and Systems Research (HPSR) at sub-national levels is also highlighted.

## Background

The health care system in India is pluralistic with the co-existence of multiple systems of traditional medicine along with biomedicine [1, 2]. The term medical pluralism was introduced in social science literature in the 1970s to describe the situation in the developing world, of people resorting to different medical systems other than biomedicine that was provided by governments [3]. Although traditional systems have had a subordinate place in the public health system following the introduction of biomedicine (also referred to as allopathic medicine), Indian systems of medicine have gained more attention in the past decade. In 2005, in its mission document the National Rural Health Mission (NRHM) initiated by the Government of India (GoI) declared the goal and strategy to ‘Revitalize local health traditions and mainstream AYUSH’. [4]. The ‘mainstreaming AYUSH’ strategy recommends integrating Ayurveda, Yoga, Unani, Siddha and Homeopathy (AYUSH) into the public healthcare system. In following the NRHM guidelines, many state governments have increased the establishment of AYUSH facilities in their public healthcare system by placing them on the same physical premises (co-located) as the biomedicine facilities [5].

National medical cultures are the product of a nation’s dominant political philosophy and the ways in which people express and find solutions to their health needs [6]. Indian historian Panikkar ([7], p.174-75) notes that in post- colonial India “the quest to revitalize indigenous medicine reflected a multipronged struggle for cultural hegemony, not only between the colonizer and the colonized, but also between different classes within the colonized society”. He observed that a large number of healers who were not literate and did not possess textual knowledge were marginalized in the process. Thus a selective professionalization took place, possibly because the Ayurveda and Unani systems are codified systems and have written documents [8–11].

While the AYUSH systems represent professionalized and codified medical systems, there also exists a widespread, largely non-codified and diverse tradition of folk systems which, in Indian policy papers are increasingly referred to as Local Health Traditions (LHT) [4, 10]. Clause 9 of the National Policy on Indian Systems of Medicine & Homoeopathy states ‘indigenous traditional medical knowledge available with the individuals, communities, tribals have not been fully tapped, documented and validated’ [12]. This clause refers to the non-codified systems of India, and states the importance of supporting these systems as well as the codified systems included under AYUSH.

LHT is a broad term that refers to home remedies and folk healers and includes the medicine systems of different tribal (indigenous) ethnic groups [10]. In this paper the term indigenous/tribal traditional medicine (abbreviated as tribal medicine) is used to refer to the medicine practiced by the traditional healers of the Khasi and Garo tribes of Meghalaya who use medicinal plants [13].

The northeast region of India has eight states that are ethnically and culturally different from the rest of India. They are largely populated by over 160 scheduled tribes or indigenous peoples ([14], p.4). The Indian government uses the term ‘tribals’ or the constitutionally recognized category of ‘Scheduled Tribes’ to refer to these communities [15].

Meghalaya state has a population of about 3 million, 86 % of whom are identified as Scheduled Tribes [16, 17]. It has a largely hilly terrain, is a biodiversity ‘hotspot’ and is home to matrilineal tribes: Khasi (and Jaintia sub-tribe) and Garo, with Khasis being the larger [18, 19]. Meghalaya has systems of tribal traditional medicine in an oral form that is largely un-documented. Most of the studies on tribal medicine conducted in the state are ethno-botanical in nature [20–23]. Despite the practice of indigenous traditional medicine being enshrined in article 24 of the United Nations declaration on the rights of indigenous peoples [24], and although informally widely acknowledged as relevant to healthcare, tribal medicine is largely ignored by the public health sector. Nevertheless, the tribal traditional healers (Khasi and Garo) are perhaps the largest group of healthcare providers in the informal sector in Meghalaya.

A health system grapples with the challenge of making services relevant to the diverse populace it serves, and medical pluralism is a policy to help address this issue. Research in health policy and health systems has evolved and become inter-disciplinary with the realisation that a linear, positivist focus on treatment and prevention of disease alone is inadequate to meet public health goals [25]. A health system is complex and needs to be adequately understood before applying measures to strengthen it. This paper presents the perceptions of tribal medicine and of AYUSH systems from various policy actors [26] in Meghalaya and locates the tribal medicine of the State within the policy of medical pluralism currently being implemented.

## Methods

The NRHM mission documents, the health statistics handbook [27], and information from the websites of the health department were drawn upon for background information on the current policies on medical pluralism being implemented in the State. Official documents, with additional information from interviews were reviewed and collated to understand the implementation of the AYUSH policy in Meghalaya and to locate the role of tribal medicine within the health policy of the state.

**Fig. 1:**
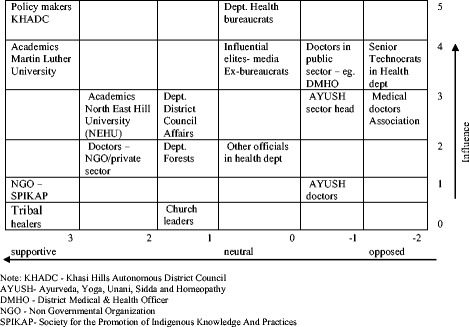
Stakeholder mapping – a matrix charting influence and support

A stakeholder mapping exercise was conducted to find an appropriate sample of policy actors [28]. The individuals and groups to be interviewed were mapped on a matrix by taking into consideration their perceived influence and power within the public health system and their interest or position on tribal medicine (Fig. 1). The development of the matrix involved subjective judgments, made in consultation with knowledgeable members of the community. The stakeholder mapping was conducted primarily as a tool to assist in sampling rather than for analysis.

Forty six in-depth interviews (Female 13, Male 33) were conducted with policy actors: bureaucrats; public servants trained in the central or state administrative services (4), technocrats; doctors with administrative duties in the Directorate of Health Services (12), policy makers of a traditional governance institution (3), biomedical doctors in the public sector (5), AYUSH doctors in the public sector (5), biomedical doctors in the non-governmental sector (4), representatives of non-governmental organizations/healer associations (3), academics (8) and elders of the community (3). Technocrats were largely allopathic doctors (9) and also AYUSH doctors (3). The labeling of roles/identities is simplified to indicate one key role of the individual. However, most individuals had more than one role and would fall into multiple categories, for instance some of the academics were also respected elders of the community. All but three of the respondents belonged to one of the ethnic tribes; Khasi or Garo.

A further 44 in-depth interviews were held with 24 Khasi and 20 Garo tribal traditional healers. Audio-recordings were made after obtaining informed consent from the participants. The qualitative data were collected between April and December 2012. Further questions and clarifications were posed to participants through email, phone and or follow-up meetings in 2013–14. Interview topic guides with relevant probing questions were piloted and developed iteratively in the field. Experiences, perceptions and attitudes to the indigenous tribal traditional system and with traditional medicines of the AYUSH systems were elicited.

The qualitative data collected were analyzed using a thematic content analysis approach that incorporated elements of grounded theory [29]. Transcripts were compared with each other, data coded, categorized and the common themes that emerged were identified. Following the grounded theory approach, line by line coding of the first set of transcripts was initiated during field work [30]. Initial analysis allowed new themes to be explored iteratively, for instance the rise in the number of AYUSH doctors in the system was not widely perceived but emerged during data analysis.

This study was approved by the institutional ethics committees of the Public Health Foundation of India and the London School of Hygiene & Tropical Medicine.

## Results

The results are presented as themes that emerged from the analysis of the qualitative data.

### The rise of AYUSH in the health system

Currently there are AYUSH facilities at the government referral hospital in the capital city, Shillong, and in public health care facilities in all the seven districts of the State. Doctors from the AYUSH systems, largely homeopathy, have been appointed to all three tiers of the health services provided by the public sector, especially Primary Health Centers (PHC). As per the government’s health statistics handbook there are 73 AYUSH treatment centers across the state in the district hospitals, community health centers (CHC) and PHCs [27]. These figures have grown since, according to personnel in the department of health, and by 2013 there were 102 co-located facilities for AYUSH across the state, 87 funded through NRHM. Most of the AYUSH services offered are of homeopathy. None of the co-located ayurveda facilities in Meghalaya currently offer *panchakarma*, key treatments in ayurveda.

The department of health was unable to provide disaggregated figures for biomedical and AYUSH doctors at the district and lower levels during the period of this study. However, information from the facility listing of doctors in the health statistical handbook ([27], p.148-165) at Primary Health Care (PHC) and Community Health Center (CHC) level indicated that there are a total of 366 doctors, including biomedical and AYUSH practitioners as well as dentists. The lack of disaggregated data for AYUSH and other doctors in the report hampered exact calculations. A more complete picture was obtained from conversations with officials in the health department. In 2012–13 there were 111 AYUSH doctors working in the public sector, of these five were based in the tertiary referral hospital in the capital. Exact figures for number of AYUSH doctors prior to the introduction of the NRHM policy were not available although there was agreement among the senior doctors interviewed that there were a few (between 10–20) homeopathic and ayurveda doctors in the state’s public sector. Thus the total number of AYUSH doctors has increased in recent years and now comprises over a quarter (29 %) of doctors in the rural doctor workforce at the CHCs and PHCs levels in the State.

Further support for the promotion of AYUSH came from allocation of funds from the government. In 2010 the North Eastern Institute of Ayurveda & Homoeopathy (NEIAH) was established in Shillong the capital of Meghalaya by the Department of AYUSH, Ministry of Health and Family Welfare, GoI. Part of its stated goal is “To generate public awareness about the potential of ayurveda and homeopathy systems of medicine for enhancing health security of rural communities including disease prevention and health promotion. To propagate ayurveda and homeopathy towards improvement of health care and mainstreaming of AYUSH systems in the region” [31]. An initial allocation of 6.75 million Indian rupees was sanctioned towards the establishment of this institution.

### Top-down approach to policy- central decisions and state level implementation

Accounts of technocrats and bureaucrats demonstrated that they were aware that the AYUSH systems were not popular in the state. The decisions to promote AYUSH in the state was based on the national policy and directives/guidelines of the Ministry of Health & Family Welfare, GoI.

Mainstreaming AYUSH [in Meghalaya] is Government of India’s [Central Government] NRHM initiative - Senior Bureaucrat, PG 028, M

To further understand how decisions that involved large financial commitments for infrastructure were made, senior technocrats who had played a role in the establishment of institutions such as the NEIAH were interviewed. Respondents agreed that they were following central directives and that states in the northeast rarely initiated these decisions. The state government depends on central funding for its health budget. The central government’s funding is a major factor in decision making as is evident from this statement from a senior technocrat in the Directorate of Health Services, GoM. He was describing the plans for setting up an AYUSH hospital in a district.

I: Okay, so is this an implementation of a central scheme rather than something which the state thought that they needed?

R: It is a Central scheme [scheme of the Ministry of Health, GoI]

I: A central scheme, and you are implementing it?

R: We are implementing it [nodding in agreement]

-Policy maker, biomedical doctor, PG 017, M

Most officials appeared to have an accepting attitude that was non-critical and unquestioning of the ‘mainstreaming AYUSH’ policy in the state. When asked why systems that are not popular locally are being promoted in northeast India, a common refrain was that the systems would become more acceptable with time, through awareness building measures. The importance of the northeast for its biodiversity and rich resource of medicinal plants were mentioned as reasons for promoting ayurveda.

Government of India wants to establish AYUSH everywhere. They want [it] to spread. […] Why Ayurveda is important here [in the northeast]? Because this is the hotspot of the biodiversity […] These decisions have been made as per [pause] because our decision is from the government of India. -Senior Technocrat, AYUSH, PG 042, M

### The role of institutionalization and competencies

For policy makers and doctors in the public sector the acceptability of AYUSH was strongly influenced by the institutionalization of these systems of medicine and the recognition provided by the central government. Institutionalization was closely linked with notions of the “scientific” authenticity of the systems. Homeopathy, which has been criticized in the medical literature [32, 33] for its lack of a scientific evidence base, was accepted as scientific by policy makers in Meghalaya.

There was a discordance noted between the competencies expected of the AYUSH doctors and what they were trained or were able to do. For example administrators were concerned by the reluctance of AYUSH practitioners to handle “emergency and serious” cases. One of the strategies advocated for utilising AYUSH practitioners as clinicians in the public sector is to train them in emergency/essential services. Following a central directive from the ministry an attempt to train AYUSH doctors as skilled birth attendants (SBA) was initiated in 2011 but was reportedly not done, partially due to a lack of acceptance of this new role among AYUSH doctors. To date no SBA training for AYUSH doctors has been conducted in Meghalaya State.

### Is AYUSH a ‘forced pluralism’ for indigenous peoples?

There was acknowledgement across all respondent groups that AYUSH systems were not well known or popular among the tribal population in the state. It was said that after biomedicine, the people preferred their own indigenous tribal traditional medicine.

There may be pockets of non-tribal populations which are familiar with ayurveda but the vast majority of the tribals are not familiar. So if one brings in an alien system of medicine it is difficult to see how the people will accept it or have faith in it. -Academic, Biomedical doctor, PG 007, M

Policy makers of the traditional governance institution, Khasi Hills Autonomous District Council (KHADC), academics and elders of the community differed in their opinions from those in the health department. These groups were concerned at the lack of support of tribal medicine in contrast to the promotion of the AYUSH systems. The notion that the introduction of AYUSH into the present format was a ‘forced pluralism’ was also alluded to as in this quote:

Whatever is introduced by the formal [public] health system, people have to accept. When a doctor prescribes, they do not know whether this is ayurvedic or this is this, they will just believe and buy that medicine. -Policy maker in KHADC, PG 004, M

AYUSH doctors shared their experiences about the lack of awareness of their systems in the community, “When they see the medicines they get in their hands, they ask what type of medicine are these? Then after we explain that these are ayurvedic medicines, they will say: no we don’t want ayurvedic medicine, we want allopathic medicine” [PG 036, F].

‘Identity’ and ‘alienation’ were themes that emerged from the narratives of elders in the community, and from academics and members of the KHADC. Their expressed support for their own system came from efficacy beliefs but also from the identification with tribal medicine as part of their culture. It was a way of exploring their own indigenous identities:

oh yes, that is there, not only with me but with many many people. Like I said it is there, there's a soft corner for, for medicinal plants and local healers. I think basically because we identify it with our own unique culture. okay, so it's something that we, we're proud of. - Academic, PG 008, M

It was also argued that if tribal medicine is dismissed without giving it a reasonable chance, potentially valuable indigenous knowledge would be lost. Inclusion and complementarity rather than exclusion of tribal medicine was the alternative suggested. They had no objections to the AYUSH streams being promoted if it was helpful to their people, but they insisted that it should not be at the cost of their own traditions of tribal medicine being neglected.

But at the same time when we have our own systems, we should see that this is not done away with, by replacing with an outside system. If it [tribal medicine] is as efficacious, it serves a certain purpose and it solves the problem of the people and in today’s world that it gives livelihood to persons I think there is every reason why we should support it. At the same time I am not saying don’t support ayurveda, don’t support homeopathy, let it be there if they serve the people, the more the merrier. -Elder, PG 002, M

### Appreciation, aversion and concerns about tribal medicine

From healer and consumer accounts it was evident that health seeking was largely divided between biomedicine and tribal medicine [13]. While many patients reportedly resorted to tribal medicine as a second option after trying biomedicine, for some illnesses it was the first option, especially in conditions that were ‘culturally bound’; wherein there was a tacit cultural understanding of the disorder.

The presence of tribal traditional healers across the state, even in remote areas, was widely accepted by policy actors. But there were divergent views on the utility of their services, with officials in the health department tending to view them as a problem, while the policy makers of the traditional institutions and others outside the health department considered them as a potential solution.

Policy actors conveyed a spectrum of opinions about tribal medicine ranging from appreciation to aversion. Those in the health department tended to refer to tribal healers as unskilled, unhygienic and unscientific. A biomedical worldview and the official role of administrators in the health department influenced their positions. For instance, those responsible for implementing programmes to reach targets on maternal and child health attributed the poor health indices to healers. However, when it came to personal needs, some officials, even those who were critical of tribal medicine, reported seeking tribal healers for their own health needs:

So since I envisaged that there was no side effect, there was no harmful effects, so why not try it. I tried and it was giving a good feeling. So why not, and it was very inexpensive at the same time - Senior bureaucrat, PG028, M

Biomedical doctors in the public sector were often skeptical, with a few being highly critical. Criticism was based on complications seen in patients who had previously used tribal medicine. While there were concerns about hygiene, for which training was suggested, almost all (except for a few doctors in the public sector) acknowledged that tribal medicine was relevant and advocated support for documentation, research, validation and the creation and building of an evidence base. The view of some doctors that most tribal healers mixed allopathic drugs into their medicinal preparation was at best a hasty generalization. Healers themselves agreed that a few who they referred to as “not genuine healers” resorted to such practices and they, as a group, were concerned about such practitioners.

The positive views of tribal medicine from policy actors outside the health department were based on their belief in the efficacy of the system that had been formed primarily through personal experiences. Comparing and contrasting their experiences with biomedicine was a method used by respondents to illustrate the efficacy of tribal medicine. Areas of tribal medicine’s expertise described as noteworthy by policy actors (which included some biomedical doctors) were treatments for burns, fractures and other musculoskeletal disorders.

### Tribal medicine and the disconnect in policy implementation

Although ‘revitalizing local health traditions’ is listed as a strategy to achieve the NRHM goals [34], it was not perceived as part of the NRHM strategy by most officials in the health department.

R: LHT [local health traditions], NRHM is not exploring at all. NRHM is not exploring LHT at all, who said they are?

I: Well in that Mission it says that [interrupted]

R: Mission is there but here [northeast India] we are not implementing - Senior technocrat, PG 042, M

Most officials in the government’s health department were unaware of the Act that was passed by the KHADC to promote and protect Khasi Traditional Medicine [35] despite the Act being gazetted and covered in the print media by all major newspapers. The KHADC is subordinate to the state government, especially with regards to health policy. Meghalaya state has not allocated any of its health budget to revitalizing its local health traditions. One argument was that there was a lack of vision in the state regarding tribal medicine. As one academic expressed: “In a sense one could say they [government] are not entirely to be blamed because when they would have a discussion about including some funding for traditional medications they’re not sure exactly where to put it: give it to the healers, set up an institute, set up a hospital for traditional medicine, and there is a perception that there is lack of data and documentation on which certain specific allocations for programmes and schemes can be based. So in that sense there are difficulties even for the government to take it up” [PG 007, M].

Thus, while on paper the stated goals of the national policy is ‘to mainstream AYUSH and revitalize local health traditions’, the latter aspect currently seems not to be a priority for the State.

### Healers as a resource- referral, livelihoods, biodiversity and indigenous knowledge

There appeared to be little direct interaction between healers and doctors in the public sector. But healers do refer patients to biomedical doctors if they are unable to help the patient. Healers pointed out that doctors were unlikely to be aware of their ‘referrals’ as such ‘referrals’ were made orally and were not accompanied by any written notes:

Yes, I do send patients to doctors and even to Shillong [capital city]. But they [doctors] do not know that we sent them because we do not give them [the patients] a slip saying that this patient has been sent by me to you for treatment, or go for an X-ray and scanning in this hospital. Khasi Healer, KH016 M, West Khasi Hills

Although doctors in the public sector failed to see reasons to engage with healers, those outside the public health sector highlighted the importance of engaging with healers for the early detection and reduction of complications. A respected psychiatrist who headed a tertiary referral institution for mental health (in the not-for-profit sector) highlighted the importance of their role. He said that his institution had as many referrals from traditional healers as they did from biomedical practitioners and explained how healers filled a gap in the existing services in mental health care using ‘depression’ as an example. He explained that healers try their own methods, often successfully, but if symptoms persisted beyond a week they usually referred their patients to allopathic doctors. A senior ophthalmologist provided accounts of early referrals from tribal healers in her institution after she conducted awareness building sessions with tribal healers. It was claimed that this helped avoid complications developing in patients with eye disorders.

Academics, elders and members of the KHADC discussed the wider benefits of tribal medicine. Healers with busy practices reported supporting the livelihoods of a network of helpers, plant collectors and suppliers. Healers were identified as stakeholders in the preservation of biodiversity and in turn their knowledge as a resource for bio-prospecting and drug development. Academics cited examples from their own research to ‘vindicate’ claims from traditional knowledge.

So, we’re looking for blueprints in plants, you know, blueprints for drugs in plants. So, traditional practitioners have been there for hundreds of thousands of years, they know the trade, they know the, its empirical knowledge, it’s through trial and error. But you see if I am to look for shortcuts, then that is where I should be looking for because that has been filtered information. I can’t, I cannot screen all the thousands of species but filtered information is a starting part. So from that perspective, I’ll give you an example… Academic PG 008, M

Thus several respondents’ particularly academics, policy makers of the KHADC and a few bureaucrats and doctors expressed the need to respect different knowledge sources and emphasised that indigenous knowledge needs to be recognised as an important resource in the state.

## Discussion

A key rationale underlying the ‘mainstreaming AYUSH’ strategy is the assumption that “The Indian systems of medicine have age old acceptance in the communities in India and in most places they form the first line of treatment in case of common ailments” [36, 37]. The degree to which different systems are supported differs from state to state within the country. State governments often preferentially promote the Indian System of Medicine that is locally popular, for example Ayurveda in Kerala and Siddha in Tamilnadu. The states of Kerala and Tamilnadu have some of the best health indices in the country, and they have been upheld as examples of providing ‘good health at low cost’ [38, 39]. This study suggests that the ‘mainstreaming’ approach being implemented in Meghalaya might have benefitted from prior health policy and systems research directed at understanding more about the use of tribal medicine in the State and the community’s preferences for different health traditions before the roll out of the national AYUSH and LHT policy. For effective delivery, health services have to go beyond the supply of ‘care’ and have to also address the acceptance and use of services [40–43].

These qualitative findings are further supported by a quantitative household survey that was conducted in all districts of Meghalaya in 2010 [13]. This study collected information on tribal medicine and AYUSH systems among rural households and demonstrated that tribal medicine was widely accepted (79.1 %) and believed to be effective (87.5 %). It was used in a wide variety of both minor and major diseases. In the 3 months prior to the survey, 46.2 reported using tribal medicine, of whom 57.9 reported cure and 33.1 % some improvement. For the AYUSH systems, a majority (68.7 %) had not heard of any of the AYUSH systems of medicine and only a minority (10.5 %) had ever used them.

### Overlooking contextual factors and the top-down approach

This implementation of central policy guidelines at the state level in Meghalaya , without enough evidence of local relevance could be seen as an example of a ‘top down’ approach to policy implementation ([44], p 128–47). Policy analysts have highlighted the importance of considering contextual factors in policy development [45]. Ideally policy ought to be tailored to the needs of the populace it seeks to serve [46, 47] if it is possible to do so. It is widely acknowledged in the policy literature that the way in which policy is implemented can differ considerably from the ideal that was intended, often referred to as the implementation gap [44]. There are numerous case studies demonstrating the poor implementation or undesirable outcomes of well -meaning policies that have been ‘imposed’ by international donors on developing countries [44]. Just as dependence on donor funding has undermined national health policy making in several developing countries [45], so within India’s federal system a state’s dependence on central funding also affect its choices. The phrase ‘forced pluralism’ had been used to describe the situation of patients with limited choices who are ‘forced’ to seek whatever is available [48, 49]. In Meghalaya it could be suggested that the state is inadvertently causing ‘forced pluralism’, albeit in a slightly different manner. But it could also be argued that providing co-located AYUSH services, even if people are not particularly interested in them, is only increasing choices for people as they are not obliged to use them if they do not wish to do so. But the question is, when people would rather have good quality biomedicine in the public sector, how relevant is this kind of pluralism?

Ignoring context can contribute to feelings of marginalization of indigenous peoples as this quote from the recently published people’s linguistic survey implies [50].

Indian intellectuals have contributed a word ‘mainstreaming’ to the English dictionary. Mainstreaming is excluding, marginalizing and demolishing smaller languages and cultures. It is another name for genocide. Tribals who are displaced from their habitats in the forests and mountains are wrenched from their languages and cultures and forced to adopt the dominant regional language as their mother tongue…

– Pattanayak, foreword in People’s Linguistic Survey of India

This is particularly relevant when one considers the socio-political climate in the northeast region. Sociologists suggest that the region is poorly understood within India; according to Karlsson [51] both geographical distance and cultural differences contribute to the misunderstanding. Tribal societies in northeast India consider themselves distinct from the non-tribals and sometimes express a sense of alienation from ‘mainland’ India [52]. Thus people in northeast cultivate a notion of otherness in their reference to the “ ‘Indian mainland’ – a place one is connected to but not really a part of ” ([51], p.49). Distinct ethnicities and the history of relative independence have led to separatist and nationalist movements which have persisted to this day ([51], p.49-61).

### LHT and AYUSH in health system strengthening

Ignorance, unsanitary habits and quackery are common prejudices about traditional healers that anthropologists have documented for decades (Leslie, 1980). Such attitudes towards tribal traditional healers were perceived and documented in this study as well among individuals within all stakeholder groups.

Biomedical doctors’ concerns about complications seen in patients who had previously used tribal medicine underline the need for systematic research, assessment and evaluation of safety and efficacy rather than hasty generalizations. The data from this study does not suggest that tribal medicine is preferable to other systems. Healers themselves acknowledged the limitations in the scope of tribal medicine and potentially some of their practices are undesirable. However, more engagement with tribal healers could for instance, lead to improved referrals for timely interventions for illnesses in which Western biomedicine has better treatment.

The classical texts of ayurveda refer to a complementary relationship between the codified knowledge and non-codified local knowledge; the *Charaka Samhita* refers to the knowledge of forest dwellers about medicinal materials [10]. The boundaries between codified and non-codified practices were more fluid in earlier periods but they became more marked after the formalizing through institutionalization [10]. Lambert [8], in her study of bone doctors in Rajasthan, argues that treatment modalities in India that would be categorized under the new terminology of LHT, have become marginalized through exclusion by the state. She observed that in “the process of formalizing medical knowledge, ‘epistemic’ expertise that can be acquired from secondary sources and tested through written examination inevitably becomes valorized over ‘performative’ expertise that is acquired through experiential learning” from oral traditions.

In India, and elsewhere in the world, it is acknowledged that indigenous and folk healers have kept alive and passed on indigenous knowledge for centuries through their oral traditions [53–57]. Clause 1.4 of the national policy states [12] “the positive features of the Indian Systems of Medicine, namely, their diversity and flexibility; accessibility; affordability; a broad acceptance by a section of the general public; comparatively low cost; a low level of technological input and growing economic value have great potentials to make them providers of health care that the larger sections of our people need”. If these systems are to become more relevant and attainable in Meghalaya state, then it is important that resources also be made available to tribal medicine. This is not to say that all their practices be accepted unquestioningly, rather that resources need to be allocated for documentation, research, engagement with healers and for appropriate assessment be performed so that the beneficial aspects of tribal medicine can be recognised. Engaging with oral traditions is hard and challenging but a start needs to be made. The Khasi Hills Autonomous District Council Act for the protection and promotion of Khasi traditional medicine [35] seems to provide an important focus to pave the way for initiating action. Further lessons can be drawn from the state of Kerala with its initiatives in the promotion of, and training in, tribal medicine [58, 59].

Trust building measures that promote dialogue with traditional healers will enable a better understanding of their role and potential contribution to the public health sector. The health department could help to create platforms to develop interactions between tribal healers and doctors in the public sector. Skill development and capacity building of Meghalaya’s vast network of tribal healers can strengthen the public health care system. They could be trained to deliver last mile services of the public health schemes and services for the government. The example of the neighbouring state of Nagaland may be useful for Meghalaya’s policy makers [60]. Nagaland has initiated training and involvement of bonesetters and traditional birth attendants into their health workforce through the NRHM.

AYUSH comprises diverse systems, each of which arises from a different epistemological and philosophical base. Gopichandran et al. [61] have highlighted some of the ethical implications in integrating these disparate systems into existing public health services. The science of ayurveda has holistic preventive and health promotive potential that moves away from an emphasis of drugs alone in treatments [62–64]. Ayurvedic treatments involve identifying disease-causing factors (*doshas*) and the restoration of the equilibrium of bodily functions and tissues using a variety of measures varying from special diets, activities, medicines and medical procedures such as *panchakarma* [62, 64–66]. For example, one aspect of prevention and treatment is the knowledge of *ritucarya* or seasonal regimens and adoption of appropriate dietary practices [66, 67]. In regions of India where ayurveda is part of the culture like Kerala, awareness of concepts such as *ritucarya* already exists within communities [67]. This potentially enables adoption of dietary regimes and non-drug based therapeutics of ayurveda more amenable to the people. The absence of such cultural understanding of relevant concepts could make the practice of ayurveda sub-optimal in Meghalaya. Of note, currently none of the co-located ayurveda facilities in Meghalaya offer *panchakarma* procedures, the therapeutic interventions that are often considered as integral part of ayurveda therapy [68, 69]. Thus it could be argued that a limited type of ayurveda is being offered in the state.

Of the AYUSH systems, homeopathy is the most widely available in Meghalaya, as is the case in several of the other northeast states [5]. It has been suggested in some reports that the benefits of homoeopathy may be compatible with the placebo hypothesis [33, 70], although admittedly the placebo effect has its benefits too and is not to be dismissed in primary health care [71, 72]. An important question that arises is what proportion of public healthcare should be provided through homeopathy.

In the report of the study conducted by the National Health Systems Resource Centre (NHSRC) to evaluate the ‘status and role of AYUSH and LHT [5] the authors acknowledge that within the Ministry of Health, GoI, there is a lack of clarity and divergent views on the primary objectives of the mainstreaming AYUSH strategy. It is viewed either as a way of securing doctors for rural areas where biomedical (MBBS qualified) doctors are not available or unwilling to be posted, or as a way of increasing access to and strengthening the services of the AYUSH systems. The data from this study suggests that the former may be the case in Meghalaya. While attempting to integrate the different AYUSH systems for universal health coverage, Patwardhan [73] recommends clear role definition for each category of doctor at the different levels of health care where knowledge, expertise and skills determine the roles rather than academic degrees. Retraining and task shifting of AYUSH doctors for alleviating shortages in human resources [74], assumes that doctors are interested in these new roles. Our data suggests that many doctors in Meghalaya are not keen on performing certain roles such as attending births and emergency medicine. A recent study to evaluate the competence of different primary health care providers in rural settings in India found the relatively new cardre of rural medical practitioners, who have a shorter (3 year) training programme, to be more competent than the AYUSH doctors at the PHC level [75]. Although the clinical vignettes used to measure clinician knowledge in that study have been critiqued for their biomedical bias [76], the study is nevertheless relevant as increasingly AYUSH doctors are expected to perform public health roles that incorporate biomedical components [77]. The effectiveness of the cardre of rural medical practitioner in providing primary health services has been further corroborated by a study from the NHSRC, and recommendations for replication of this model have been made [78]. Our study indicates people’s preference for biomedicine within the public health facilities in rural Meghalaya. The approach of the neighboring state of Assam, to train and engage a specific cadre of rural medical practitioners in a 3 year training programme [75], is perhaps an example that Meghalaya needs to explore to help it to address its shortages of human resources in the health system in rural areas [79].

In the NHSRC 18 state study, among the five northeastern states in the study: Assam, Manipur, Tripura, Nagaland and Sikkim, disaggregated data on utilization of AYUSH and allopathy services was available only from Nagaland, where AYUSH formed 4 % of outpatient use (p. 84). The study recommends increasing AYUSH services in all the northeast states except Tripura and Manipur ([5], p.xxxi). The 12th Five Year Plan recommends “in case a facility does not attract expected case-loads, the staff may be rationalised” ([74], p.35). Undoubtedly the sustained promotion of the AYUSH systems will improve utilization as is evident from the recent 7^th^ Common Review Mission report from Meghalaya. Outpatient numbers demonstrate how the relative proportion of AYUSH utilization over the years has increased from 3.9 in 2009–10 to 4.3 in 2011–12, to 5.6 in 2012–13 and to 6.5 in 2012–13 [80]. Thus there is a marginal increase by about a percentage point each year. However, it is uncertain whether this is the most effective or efficient means of providing good quality primary health care that is agreeable and resonant with local community preferences with different systems of medicine.

### Paucity of health policy and health systems research at the sub-national level

There is a paucity of health systems research from Meghalaya and other parts of northeast India. The significance of the role of research and policy analysis in health systems strengthening has been well articulated [45, 47, 81, 82]. Health Policy and Systems Research has been described as a field that is driven by questions that arise from the ground [83]. If attention is paid to defining the problem and framing the right research question, more efficient health systems can emerge [84–86]. According to a WHO document on systems thinking, even simple interventions targeting one area of a health system can have counter intuitive effects elsewhere in the system [87]. Many health systems in low and middle income countries lack the capacity to measure or understand their own weaknesses and constraints. Measuring the effectiveness of multi-faceted and complex interventions is difficult in these countries and approaches to evaluation are often weak or entirely absent [87].

Increased deployment of the AYUSH cadre of practitioner in Meghalaya has taken place alongside the introduction of other health system strengthening measures such as providing a network of emergency ambulance services for rural areas. These services are likely to have directly benefited the community. In the absence of research, improvements in health indices will be attributed to all the health systems strengthening measures that were employed. But to extend this conclusion to the mainstreaming AYUSH strategy without sufficient disaggregated evidence would be misleading. Likewise, research and documentation of the concepts and practices in tribal medicine, its efficacy, contribution to indigenous knowledge, biodiversity preservation and livelihoods also need to be considered urgently. This study contributes to the under- researched areas of context, and implementation of policies on medical pluralism. But it has several limitations for instance although efforts were made to include a broad group of stakeholders in order to capture diverse views, some groups have been left out, such as elected representatives to the state legislative assembly. This was a small sample that cannot claim to be representative of the entire state.

## Conclusions

Promotion of AYUSH in the public sector has led to a significant increase in AYUSH, especially homeopathic, doctors in the public health system in rural Meghalaya. While this has improved the ratio of health care providers, the low awareness and understanding of the AYUSH systems raises questions on how these systems should be promoted in the tribal communities when the community’s preference is for biomedicine and their own tribal medicine. There has been little support extended to tribal medicine in the state despite the widespread presence of healers and the extensive use of their services within the communities. The popularity of tribal medicine, along with the perceived benefits expressed by a large proportion of the stakeholders, plus the wider implications of livelihoods, biodiversity preservation, indigenous knowledge and bioprospecting, suggests that tribal medicine needs to be supported. By engaging with tribal healers the public sector could promote learning, help to dissuade inappropriate practices, and enable documentation and improved referral, which in turn would contribute to the overall strengthening of the health system. This study highlights the importance of contextualizing the policy of medical pluralism in the culture of the State and the need to conduct health systems research at the local level to provide evidence to inform health policy formulation.

## Abbreviations

AYUSH: Ayurveda, Yoga, Unani, Sidda and Homeopathy
CHC: Community Health Centre
GoI: Government of India
GoM: Government of Meghalaya
KHADC: Khasi Hills Autonomous District Council
LHT: Local Health Traditions
NFHS: National Family Health Survey
NEIAH: North Eastern Institute of Ayurveda & Homoeopathy
NHSRC: National Health Systems Resource Centre
NRHM: National Rural Health Mission
PHC: Primary Health Centre
SPIKAP: Society for the Promotion of Indigenous Knowledge and Practices
WHO: World Health Organization
